# Book Notices

**Published:** 1882-10-20

**Authors:** 


### *BOOK NOTICES.*

THE INTERNATIONAL ENCYCLOPEDIA OF SURGERY, a Systematic Treatise on the Theory and Practice of Surgery, by Authors of Various Nations, Edited by John Ashurst, Jr., M.D., Professor of Clinical Surgery in the University of Pennsylvania. Illustrated with Chromo-Lithographs and Wood Cuts. In Six Volumes. Vol. II. New York: William Wood & Co. McGarrity & Laird, Agents, Atlanta, Ga. 1882.

This, the second volume of the above great work, is a book containing 754 octavo pages. It is beautifully illustrated with colored plates and contains a vast amount of surgical information, ably presented and well up with modern advances and the most improved methods. "The present (or second) volume opens with articles upon those affections, such as Wounds, Burns, Abscesses and Gangrene, which, though local in themselves, may be met with in any region of the body. Then follow elaborate articles upon the various Venerial Diseases: Gonorrhea, the Simple Venerial Ulcer or Chancroid, Syphilis, Vegetations, etc.; and in the latter part of the volume is begun the consideration of Injuries and Diseases of the various Tissues of the Body."

FISTULA, HEMORRHOIDS, PAINFUL ULCERS, STRICTURE, PROLAPSUS, and other diseases of the Rectum—their Diagnosis and Treatment. By Wm. Allingham, M. D., Fellow of the Royal College of Surgeons of England; Surgeon to St. Mark's Hospital for Fistula and other Diseases of the Rectum, etc. Fourth revised and enlarged edition. with illustrations. Philadelphia: P. Blakiston, Son & Co. 1882.

This work contains 165 octavo pages and much valuable and practical information little understood and greatly needed by many practitioners.

